# Mapping the research landscape of minor cannabinoids: a bibliometric analysis of research trends and hotspots

**DOI:** 10.1186/s42238-026-00402-2

**Published:** 2026-02-10

**Authors:** Hanane Abbou, Lahcen Belyamani, Rachid Eljaoudi

**Affiliations:** 1 Mohammed VI University of Sciences and Health (UM6SS), Casablanca, Morocco; 2 Mohammed VI Center for Research and Innovation (CM6RI), Rabat, Morocco; 3 Biotechnology lab (MedBiotech), Bioinova Research Center, Medical and Pharmacy School, Mohammed V University in Rabat, Rabat, Morocco; 4 Department of Emergency, Mohammed V Military Training Hospital, Mohammed V University of Rabat, Rabat, Morocco

**Keywords:** Minor cannabinoid, Bibliometric analysis, Cannabis, Research landscape

## Abstract

**Background:**

Minor cannabinoids, including cannabigerol (CBG), cannabinol (CBN), and cannabichromene (CBC), are gaining scientific attention for their distinct therapeutic potential beyond THC and CBD. Despite this growing interest, research on these compounds remains fragmented and underrepresented in the literature. This study aims to map the global research landscape of minor cannabinoids through bibliometric mapping analyses, identifying key trends, collaboration patterns, and emerging thematic areas.

**Methods:**

A structured database search of the Scopus, PubMed, and WOS databases identified 1516 eligible articles published between 1969 and 2024. An inclusive search strategy was employed to capture minor cannabinoid research as it is embedded within the broader cannabinoid literature, ensuring that studies co-analyzing major and minor cannabinoids were not overlooked. Bibliometric analysis was conducted using the Biblioshiny tool to assess publication trends, journal impact, geographic distribution, and author collaboration networks.

**Results:**

Publication activity showed exponential growth starting in 2017, reaching a peak in 2024. The United States, Italy, and Canada led global contributions, with widespread international collaboration. Core publishing venues included Molecules, The *Journal of Analytical Toxicology*, *the British Journal of Pharmacology*, and *Cannabis and Cannabinoid Research*. Keyword co-occurrence analysis revealed three major thematic clusters. A sharp thematic shift has been observed since 2015, highlighting the rise of **“**CBG”, “CBGA” and “molecular docking”. This evolution marks a convergence of traditional pharmacology with molecular targeting and bioinformatics, signaling a transition toward computational and receptor-targeted research.

**Conclusions:**

Minor cannabinoid research is expanding rapidly, with strong interdisciplinary foundations and growing global collaboration. This study provides a comprehensive overview of the field’s evolution and highlights underexplored areas ripe for future investigation.

**Supplementary Information:**

The online version contains supplementary material available at 10.1186/s42238-026-00402-2.

## Background

In recent years, the scientific community has witnessed a surge in interest in minor cannabinoids, which were once overshadowed by their more prominent counterparts, such as Δ⁹-tetrahydrocannabinol (THC) and cannabidiol (CBD) (Caprioglio et al. 2022). These lesser-studied compounds, collectively referred to as “minor cannabinoids,” include cannabigerol (CBG), cannabinol (CBN), cannabichromene (CBC), and others, and have begun to make significant contributions to various fields of medicine and pharmacology.

The term “minor” refers primarily to the relatively low concentrations of these compounds found in cannabis plants compared to THC and CBD; however, this nomenclature does not reflect their potential significance in therapeutic applications. Indeed, research has shown that some minor cannabinoids possess unique properties that could be harnessed for treating conditions ranging from epilepsy and obesity to pain management and dermatological disorders (Wong and Cairns 2019; Walsh et al. 2021; Kwiecień and Kowalczuk 2023).

Despite the growing body of evidence supporting the therapeutic potential of minor cannabinoids, the scientific literature on these compounds remains sparse and fragmented. This scarcity is partly due to historical regulatory restrictions and the challenges associated with isolating and synthesizing these compounds in sufficient quantities for comprehensive study (Caprioglio et al. 2022).

However, advancements in analytical techniques, such as mass spectrometry and high-performance liquid chromatography (HPLC), along with increased global acceptance of cannabis research, pave the way for deeper exploration of minor cannabinoids’ molecular pharmacology and clinical applications (Pacifici et al. 2017; Citti et al. 2018; Tolomeo et al. 2021).

The present study aims to provide an overview of the current scientific landscape of minor cannabinoid research, identifying the hotspots of investigation and the unexplored areas that warrant further attention. To achieve this, we will employ bibliometric analysis to review the existing literature and analyze trends in this field.

Using these methodologies, we seek to highlight emerging trends, key themes, and potential future directions in this rapidly evolving field. Understanding the interplay between different research domains, such as chemistry, biology, and clinical applications, will be crucial for advancing knowledge and translating findings into practical therapies.

## Methods

### Data source and search strategy

To ensure a comprehensive review of the literature on minor cannabinoids, we utilized a multi-database search strategy, which included Scopus, Web of Science, and PubMed as our primary sources for document retrieval (Abdullah et al. 2024). The final search was conducted on December 10, 2025, across all three databases.

Our search strategy was designed to be inclusive, avoiding restrictive exclusionary filters. This approach captures minor cannabinoids not only as isolated subjects but also where they are investigated alongside CBD and THC. Consequently, the resulting dataset represents minor cannabinoid research embedded within the broader cannabinoid literature, providing a holistic view of the field. The search equations employed in Scopus, Web of Science, and PubMed are detailed in the supplementary material (S1). These queries were structured to include terms related to various minor cannabinoids and their derivatives, ensuring broad coverage of relevant topics. Data were exported in csv format, ensuring all metadata fields (including full record, cited references, and full author names) were retained to minimize author ambiguity. Duplicate records were identified and removed manually using Microsoft Excel by checking for duplicate Digital Object Identifiers (DOIs) across the datasets.

### Data screening and management

Data management was conducted using Microsoft Excel. The study design is defined as a quantitative bibliometric analysis and conceptual structure mapping. To ensure the transparency and reproducibility of the data selection process, we adopted the reporting standards of the PRISMA 2020 (Preferred Reporting Items for Systematic Reviews and Meta-Analyses) statement (Page et al., [Bibr CR29][Bibr CR28]). PRISMA guided our data screening and selection process to ensure the inclusion of high-quality, relevant studies while minimizing bias and enhancing reproducibility. The PRISMA framework was employed strictly as a guideline for the bibliographic identification and screening phases; as this study focuses on mapping scientific production rather than synthesizing clinical evidence, no protocol registration or risk-of-bias assessment was performed (Adawiyah et al. 2023; Azizan 2024a).

Data screening was performed by the lead author. As this study is a bibliometric analysis rather than a systematic review of clinical interventions, screening was conducted based on objective bibliographic metadata to ensure topical relevance. The final dataset was established based on the following inclusion and exclusion criteria: records were included if they were original research articles or reviews, published in the English language up to 2024, and topically relevant to minor cannabinoids as the primary focus. Records were excluded if they were conference papers, book chapters, errata, letters, or studies primarily focused on THC or CBD with only incidental mention of minor cannabinoids.

The dataset was manually inspected for missing metadata (e.g., missing publication years or author names). Where possible, missing values were manually retrieved from the publisher’s website and corrected in the dataset before analysis.

### Data analysis

To comprehensively analyze the scientific landscape of minor cannabinoids research, we employed a multi-faceted approach utilizing R software (v.4.4.1) and the Bibliometrix package (v.4.3.0) via the Biblioshiny interface (Aria and Cuccurullo 2017; Azizan 2024b). This approach follows best practices in bibliometric science mapping, including those outlined in Donthu et al. (2020) (Donthu et al. 2020).

We assessed annual scientific production to track growth trends over time and evaluated journal performance based on the total volume of articles published. At the geographic level, international collaboration was mapped using a social structure network analysis based on all author affiliations. The network was constructed to visualize the global flow of knowledge, using total link strength to quantify the intensity of joint research efforts between nations. To ensure the visualization focused on significant and sustained partnerships, the network was pruned by setting the minimum node degree to 2 and the minimum edge weight to 5.

To accurately reflect the volume of participation in international research networks, we also employed full counting for the collaboration analysis, assigning full credit to each country affiliated with a publication. Foundational works were identified through citation analysis based on Total Global Citations. Furthermore, to investigate research hotspots and conceptual structures, we applied bibliometric text mining techniques, including tokenization and stop-word removal, on Keywords Plus metadata. A keyword co-occurrence network was constructed using the top 100 most frequent keywords; this network was normalized using the Association Strength index (van Eck and Waltman 2009), and distinct thematic research communities were identified using the Walktrap clustering algorithm (Lancichinetti and Fortunato 2009), while the minimum node degree was set to five. Finally, temporal analysis of high-frequency keywords was conducted to visualize the evolution of research themes by mapping the frequency distribution of keywords over time.

By combining these methodologies, we aimed to provide a holistic view of the current state and future directions of minor cannabinoid research, identifying both established hotspots and underexplored areas worthy of further investigation.

## Results

### Descriptive statistics

**Fig. 1:**
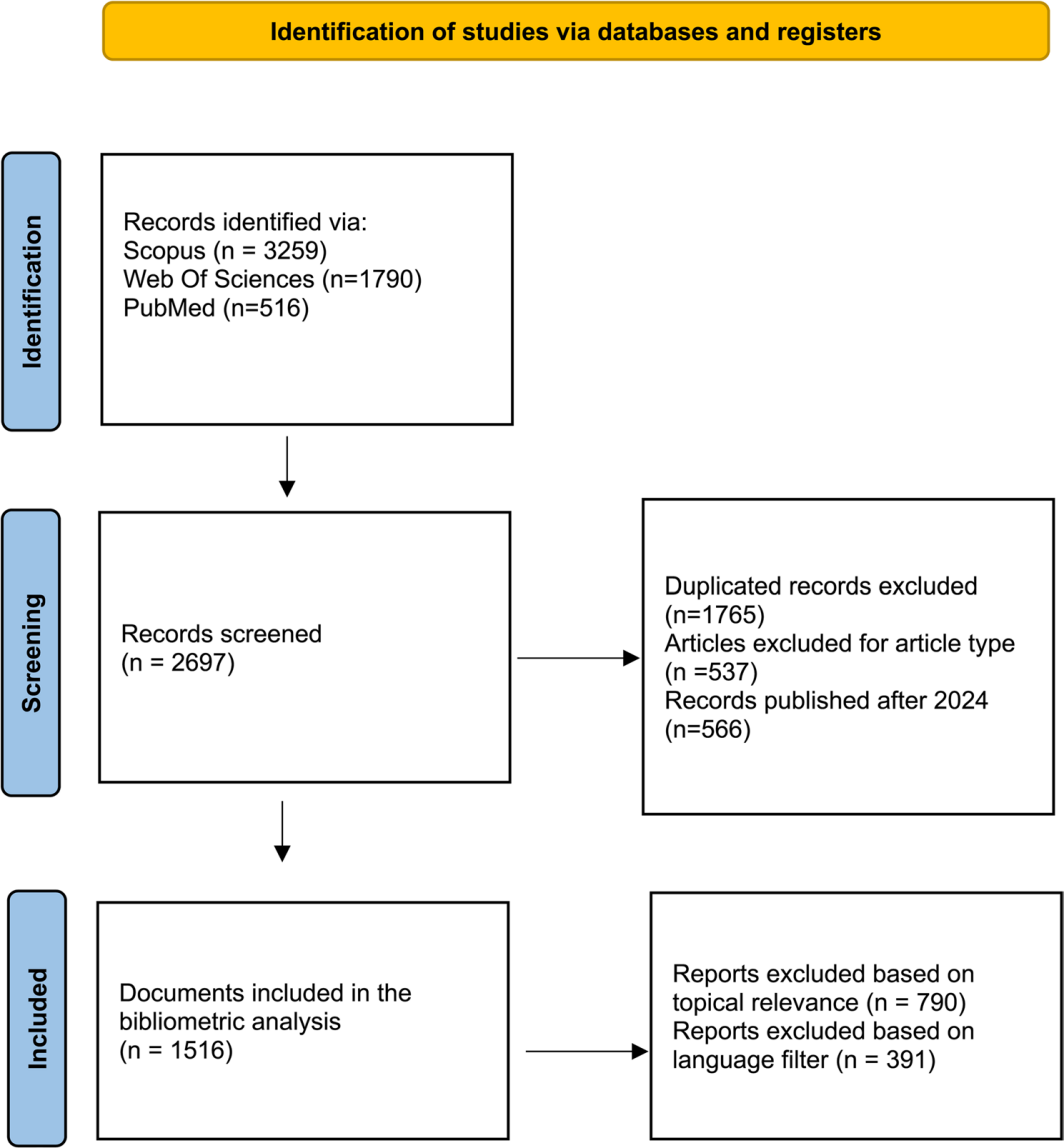
PRISMA chart illustrating the filtration and selection process of articles considered for the scientometric analysis

The initial search yielded 5565 original research articles and reviews, of which 1765 were duplicate records. The subsequent eligibility assessment filtered out 2284 records, resulting in a pool of 1516 records considered for the bibliometric analysis (Fig. 1).

### Publication growth

The annual publication growth curve reveals a clear and accelerating trajectory in research focused on minor cannabinoids, reflecting the growing scientific and societal interest in this emerging field. Between 1969 and 2017, publication activity was sparse, with most years seeing fewer than four articles and several years with none at all, suggesting an early exploratory phase or limited recognition of the significance of minor cannabinoids. Only 608 articles were published during these 49 years. Over the entire 55-year period, 1516 articles were published, with notable momentum building around 2010, when 24 articles were published, followed by a period of steady growth throughout 2018.

A major inflection point occurred in 2018, initiating a rapid surge in output that culminated in a peak of 157 publications in 2024, representing a remarkable 7750% increase compared to 1969, when just two articles were published. This sharp rise in scholarly activity likely reflects heightened academic and public interest, spurred by shifting societal attitudes toward cannabis, advances in analytical and biosynthetic methods, greater regulatory openness, and expanding investment in cannabinoid-based innovations (Kvillemo et al. 2022; Ransing et al. 2022; Siddiqui et al. 2022; Hossain and Chae 2024).

**Fig. 2:**
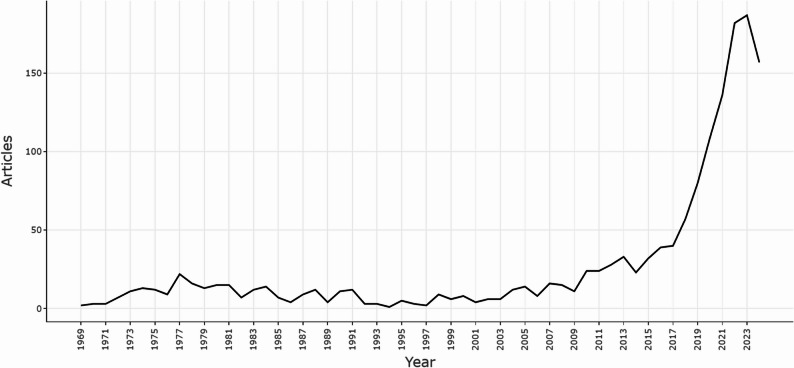
Annual scientific production of articles related to minor cannabinoids between 1969 and 2024

The upward trend also underscores the increasing recognition of the therapeutic potential of minor cannabinoids across diverse fields, including medicine, pharmacology, and neuroscience (Leinen et al. 2023; Cammà et al. 2025) (Fig. 2).

### Journals and publishing patterns

The distribution of articles across journals provides insight into the most prominent and specialized platforms for research on minor cannabinoids. Among the journals, *Molecules* leads with 45 articles, making it a significant contributor to this field. This journal focuses on biochemistry, organic chemistry, pharmacology, antioxidants, and food science, which aligns well with the interdisciplinary nature of cannabinoid research.

The *Journal of Analytical Toxicology*, with 37 articles, is another key player in publishing research in natural compounds, including minor cannabinoids. This journal has historically been a valuable source for studies underscoring detection methodologies and biological matrices. Additionally, *the British Journal of Pharmacology* and *Cannabis and Cannabinoid Research*, both of which feature 35 articles, highlight the field’s dual emphasis on rigorous pharmacological characterization and specialized cannabis-centric investigation.

**Fig. 3:**
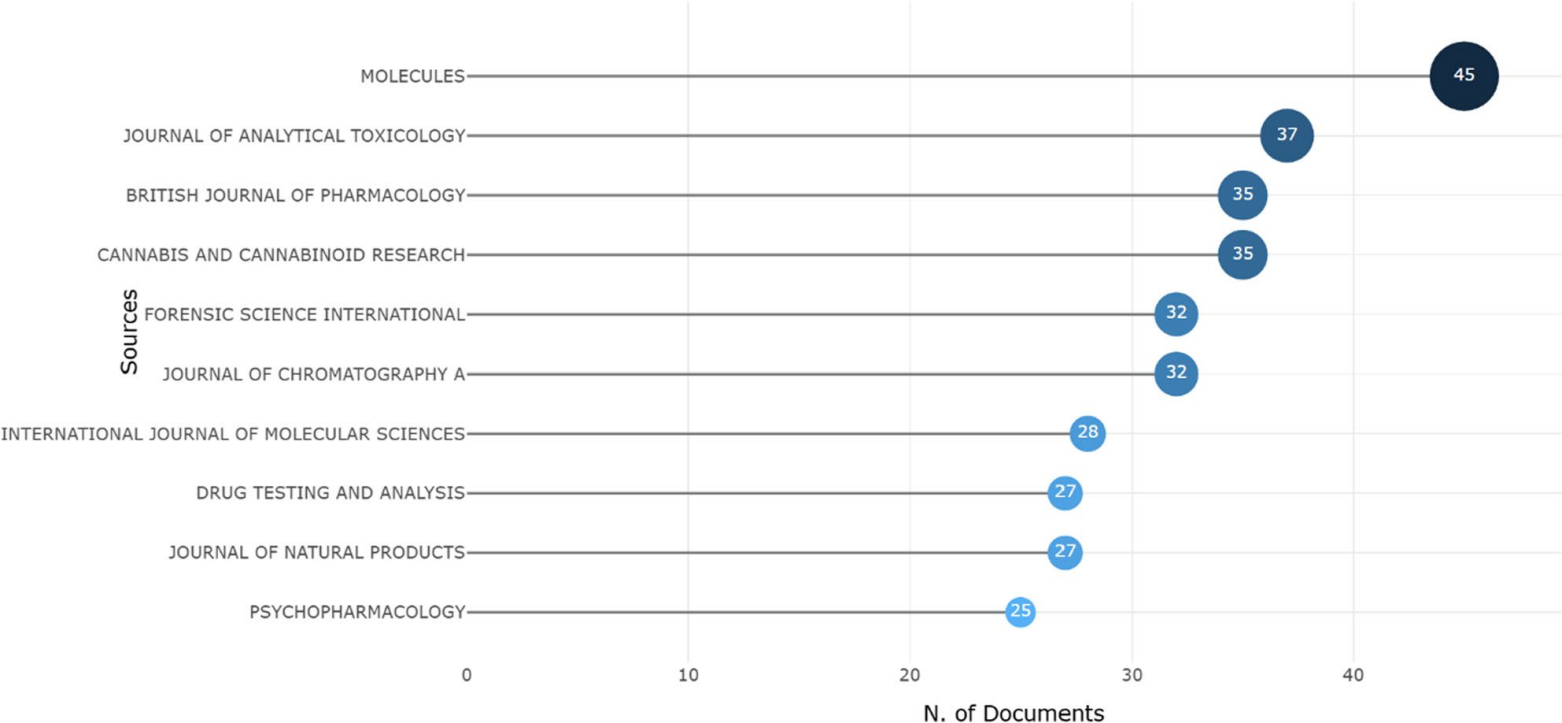
Graphical representation of the Top 10 most productive journals in the field of minor cannabinoids

Furthermore, the prominence of *Forensic Science International* and the *Journal of Chromatography A* (each with 32 articles) indicates a robust research domain dedicated to the separation, identification, and legal analysis of these compounds. Multidisciplinary and natural product research remains vital, with the *International Journal of Molecular Sciences* (28 articles) and *the Journal of Natural Products* (27 articles) providing key platforms for studies on molecular mechanisms and compound isolation. Other notable contributors include *Drug Testing and Analysis* (27 articles) and *Psychopharmacology* (25 articles), which illustrate the expanding reach of the field into behavioral sciences and regulatory testing (Fig. 3).

In summary, these results highlight not only the diversity but also the growing importance of minor cannabinoid research across various scientific disciplines, published in both specialized and high-impact journals. This wide distribution underscores the need for continued exploration and collaboration among researchers worldwide. 

### Country Scientific Production and Collaborations

**Fig. 4:**
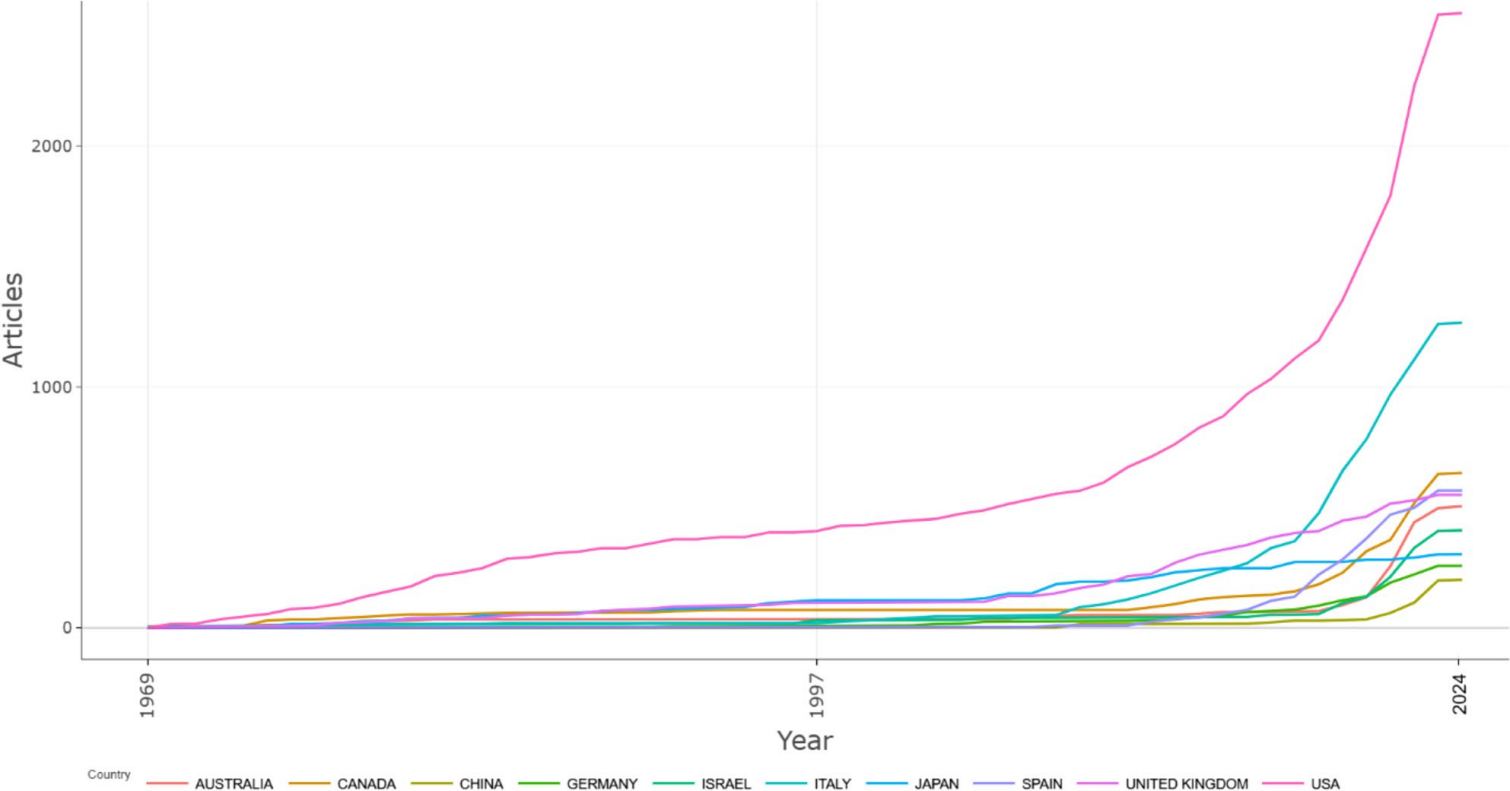
Cumulative growth of scientific publications from the top 10 most productive countries (1969–2024)

At the country level, the United States, Italy, and Canada emerged as the leading countries, with strong research contributions in the field (Fig. 4).

**Fig. 5:**
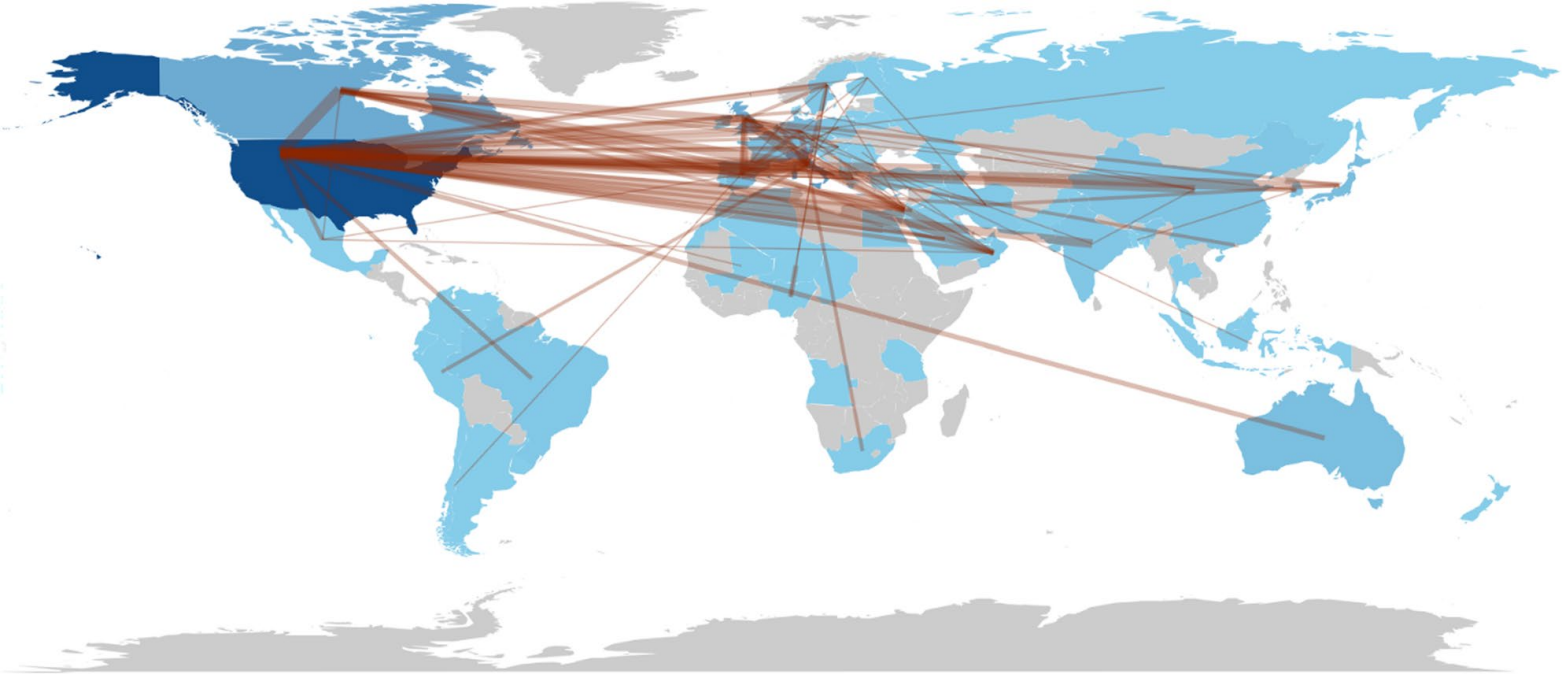
International collaboration network on minor cannabinoids. Country shading corresponds to publication volume, where darker tones indicate higher scientific production. The thickness of connecting lines (edges) represents the intensity of collaboration (Total Link Strength) between nations. To enhance visual clarity and highlight robust partnerships, the map displays only countries and links meeting a pruning threshold of minimum edge weight = 5

An analysis of the international collaboration Network (Fig. 5) indicated that cross-border partnerships were particularly prevalent, with frequent co-authorship links between researchers in North America, Europe, and Asia.

### Keywords co-occurrence analysis

The dataset comprises Keywords Plus terms extracted from publications related to minor cannabinoids. Out of the analyzed articles, 161 records lacked these metadata. A manual search was conducted, but the metadata could not be completed; therefore, they were removed from subsequent analysis.

The Walktrap algorithm optimized network modularity to partition the top 100 keywords into three distinct clusters based on co-occurrence patterns. This analysis revealed a well-structured conceptual landscape, where each cluster was interpreted according to the semantic dominance of its constituent terms. Cluster 1 (red) represents the largest and most integrative domain, centering on Clinical Pharmacology and Specific Cannabinoids. It encompasses prominent keywords such as “CB2,” “cannabidiol,” “dronabinol,” “CBN,” “CBG,” and “human.” This cluster reflects the translational bridge between specific molecular targets (particularly the CB2 receptor) and clinical investigations in human subjects. Cluster 2 (blue) relates to Preclinical and Experimental Models, anchored by terms such as “nonhuman,” “animal,” “mice,” “rat,” and “in vitro study.” These keywords emphasize the foundational research investigating physiological mechanisms and drug effects in cellular and animal models. Cluster 3 (green) comprises keywords associated with Analytical Chemistry and Forensic Toxicology, including “liquid chromatography,” “tandem mass spectrometry,” “limit of detection,” and “substance abuse detection.” This cluster highlights the methodological backbone required for the precise quantification and identification of these compounds in complex biological matrices (Supplementary Table S2).

**Fig. 6:**
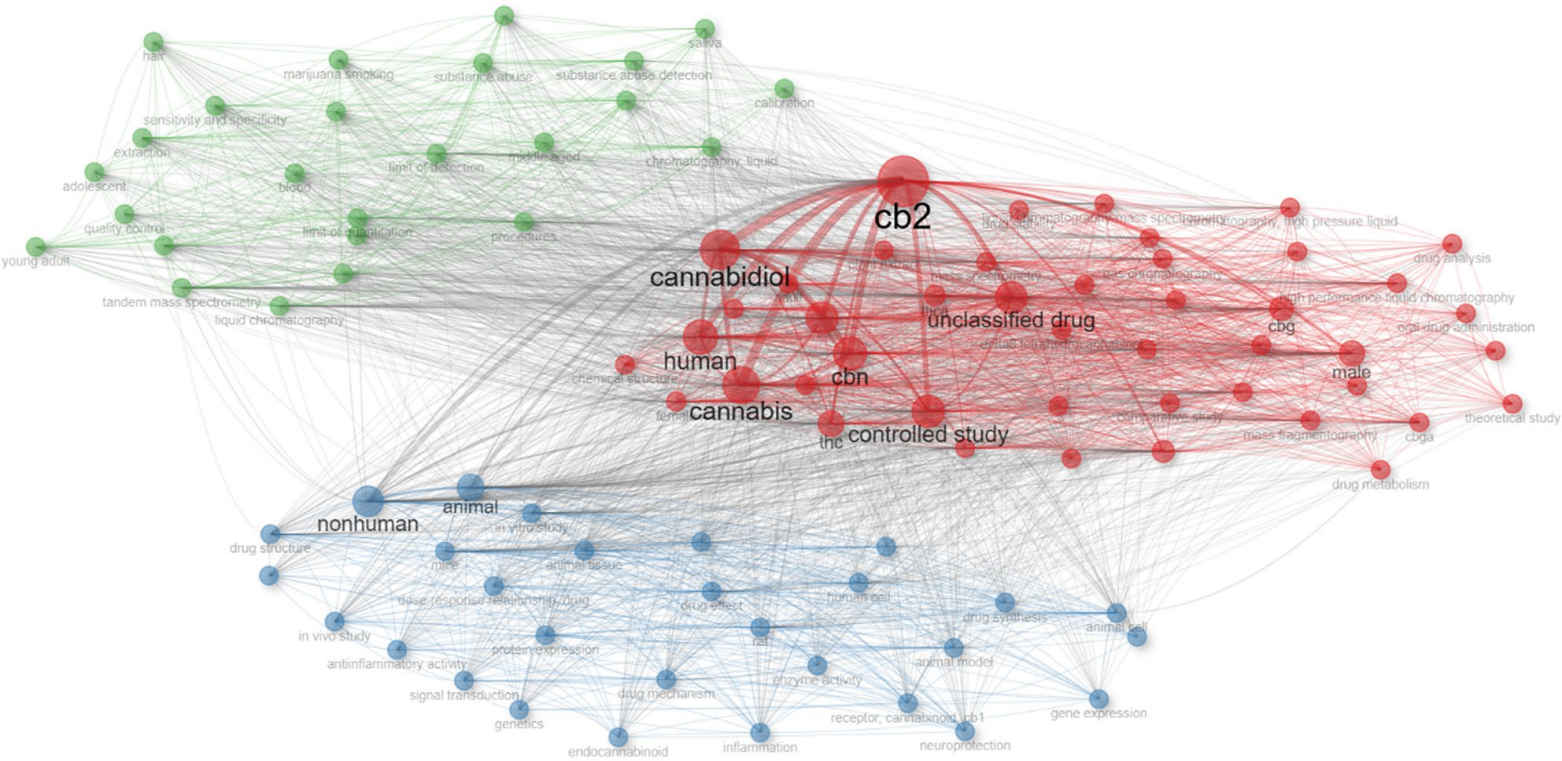
Co-occurrence network of the top 100 Keywords Plus. The map employs Association Strength normalization and Walktrap clustering (min. degree = 5), with node size proportional to frequency. See Supplementary Table S2 for the complete list of keywords and clusters

The terms “cannabis”, “human”, and “CB2” emerged as the most central nodes in the network, indicating their critical roles in linking disparate thematic areas. Notably, “CB2” served as the principal hub, reflecting its semantic and conceptual centrality in the field. The interconnected nature of the clusters underscores the multidisciplinary structure of minor cannabinoid research, integrating molecular sciences, pharmacology, neuroscience, and analytical chemistry (Fig. 6).

### Temporal evolution of research themes

**Fig. 7:**
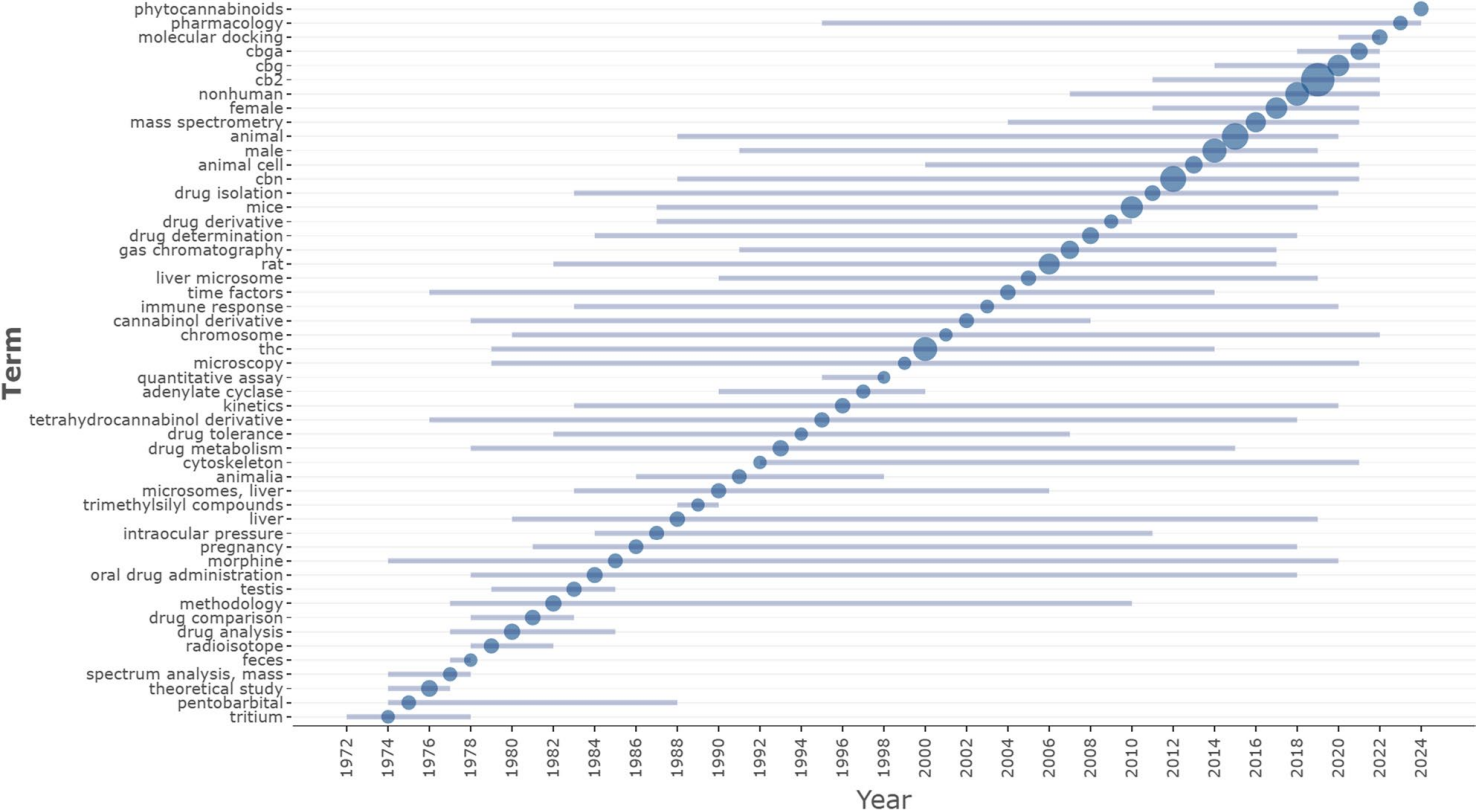
Temporal trends of high-frequency Keywords Plus (1969–2024). This visualization maps the emergence and persistence of dominant research themes over time based on Keywords Plus. The horizontal line segments represent the time span during which a term appeared with significant frequency, while the bubble size is proportional to the keyword’s frequency in that specific year

The temporal analysis of Keywords Plus reveals a clear thematic evolution in the research landscape over the past five decades, moving from foundational metabolic studies to advanced receptor-focused and computational research. Early topics, spanning the 1970 s through the 1990 s, were heavily centered on pharmacokinetics and metabolism, with prominent terms such as “drug metabolism,” “kinetics,” “microsomes, liver,” and “radioisotope”. This indicates that the foundational era of minor cannabinoid research was primarily concerned with understanding how these compounds are processed and metabolized by biological systems, often using “tritium” and “pentobarbital” as experimental standards. By the 2000 s and early 2010 s, the focus shifted toward molecular and methodological precision, characterized by the emergence of terms like “chromosome,” “immune response,” and “gas chromatography” (2001–2007). This transitional period marked a growing interest in the genetic basis of cannabinoid effects and the refinement of isolation techniques necessary to separate minor compounds from the major phytocannabinoids (Fig. 7).

A sharp thematic paradigm shift is observed in the modern era (2015–2024), where research has moved decisively toward specific molecular targets and in silico modeling. “CB2” (cannabinoid receptor 2) emerged as the dominant topic of this period (median year 2019), appearing with the highest frequency in the dataset, reflecting a concerted effort to explore non-psychotropic therapeutic targets. This was immediately followed by a surge in research on specific minor cannabinoids and their precursors, with “CBG” (cannabigerol) peaking in 2020 and “CBGA” (cannabigerolic acid) in 2021. Most notably, the appearance of “molecular docking” in 2022 and “phytocannabinoids” in 2024 highlights the field’s latest integration of bioinformatics and structural biology. These trends suggest that a convergence of high-precision pharmacology, specific receptor targeting, and computational simulation defines the current frontier of minor cannabinoid research.

### Most globally cited publications analysis

The most cited document analysis highlights the foundational and high-impact contributions that have shaped the scientific discourse on cannabinoids and cannabis-related pharmacology. The top-cited paper is “*The diverse CB1 and CB2 receptor pharmacology of three plant cannabinoids: Δ9-tetrahydrocannabinol*,* cannabidiol and Δ9-tetrahydrocannabivarin*” (Pertwee 2008), published in the *British Journal of Pharmacology* (1660 citations). This seminal review marks a cornerstone in minor cannabinoid research by rigorously defining the receptor profiles of THCV (tetrahydrocannabivarin) alongside major cannabinoids. Its dominant citation count reflects its role as the primary reference for understanding the specific pharmacological mechanisms, agonism, antagonism, and inverse agonism, that distinguish minor cannabinoids from their psychoactive counterparts.

The second most-cited paper, *“Pharmacokinetics and pharmacodynamics of cannabinoids”* (Grotenhermen 2003) (1220 citations), published in *Clinical Pharmacokinetics*, provided the first comprehensive establishment of the metabolic and bioavailability profiles of phytocannabinoids in humans. Its sustained high impact underscores the field’s long-standing focus on the physiological processing of these compounds, which remains critical for the development of standardized therapeutic formulations.

The third most cited article, “*Cannabis sativa: The Plant of the Thousand and One Molecules*” (Andre et al. 2016) (1198 citations), published in *Frontiers in Plant Science*, represents the modern integrative era of cannabis science. Unlike earlier pharmacological studies, this work bridges phytochemistry and botany, offering an exhaustive inventory of the diverse secondary metabolites in cannabis, including the full spectrum of minor cannabinoids, terpenes, and flavonoids. Its prominence aligns with the bibliometric trend towards the “entourage effect” and the holistic characterization of the plant’s chemical diversity.

**Fig. 8:**
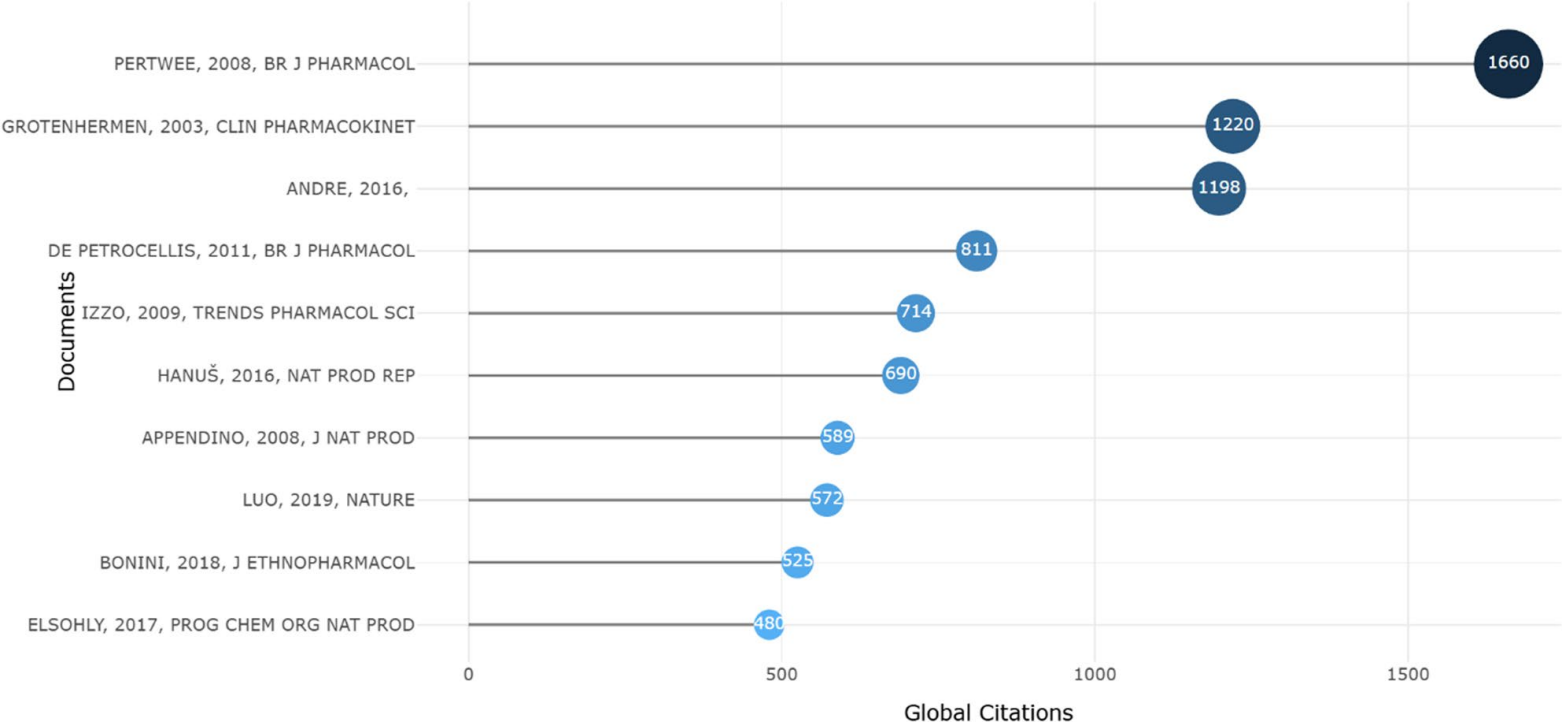
Graphical representation of the Top 10 most cited documents related to minor cannabinoids. The documents are ranked based on “Global Citations”, which is the total number of citations each article has received as of the date of data extraction

Collectively, these documents serve as intellectual cornerstones, bridging receptor pharmacology (Pertwee), clinical pharmacokinetics (Grotenhermen), and botanical chemistry (Andre). The presence of other highly cited works, such as De Petrocellis (2011) (811 citations) (De Petrocellis et al. 2011) and Izzo 2009 (714 citations) (Izzo et al. 2009), further reinforces the strong influence of experimental pharmacology in bridging the gap between natural product chemistry and clinical application (Fig. 8).

## Discussion

This study presents a comprehensive bibliometric analysis of global research trends related to minor cannabinoids, providing insight into both the historical evolution and the current thematic structure of the field. It is important to note that the dataset reflects minor cannabinoid research embedded within the broader context of cannabinoid science. The presence of major cannabinoids (e.g., in keyword clusters) is a consequence of the inclusive search strategy employed to capture the full scope of minor cannabinoid investigations. The increasing volume and diversification of research over the past two decades highlight the growing scientific, medical, and regulatory interest in this previously underexplored class of bioactive compounds.

The sharp growth in publication output, particularly from 2017 onward, temporally aligns with an inflection point in cannabinoid science. This surge coincides with several converging factors, including the legal reclassification of cannabis and hemp in multiple jurisdictions, increased public demand for plant-based therapeutics, and growing interest from the pharmaceutical sector (Russo 2019; Ransing et al. 2022; Chaachouay and Zidane 2024). While bibliometric data cannot establish a direct causal link, the synchronicity between these legislative milestones and the surge in academic output suggests a strong association between regulatory openness and scientific productivity. Similarly, the rise in analytical publications parallels advancements in high-resolution mass spectrometry, suggesting that technological availability has been a key enabler of compound identifications (Omar et al. 2013; Hazekamp 2018; Olejar et al. 2022).

The analysis of publishing patterns reveals that the field is multidisciplinary by nature, drawing contributions from molecular sciences, pharmacology, plant biology, and analytical chemistry. Journals such as Molecules, the *Journal of Analytical Toxicology*, the *British Journal of Pharmacology*, and *Cannabis and Cannabinoid Research* have emerged as key platforms, reflecting a balance between fundamental chemical characterization and applied biomedical research. The presence of specialized outlets like *Journal of Analytical Toxicology* and *Forensic Science International* suggests a bibliometric maturation of the field, moving beyond botanical exploration toward rigorous detection and toxicological characterization.

Geographically, the research landscape is dominated by the United States, Italy, and Canada, consistent with earlier bibliometric findings that highlight these nations’ leadership in both natural product chemistry and translational cannabis research (Haboubi et al. 2024; Laaboudi et al. 2024). The prevalence of international collaborations, particularly among researchers in Europe and North America, signals a growing global network that fosters knowledge exchange and accelerates discovery. The high number of collaborations is also reflective of the need for constant beneficial exchange between high cannabis producing countries and scientifically advanced countries.

Keyword co-occurrence analysis further reinforces the interdisciplinary complexity of the field. The emergence of three major conceptual clusters, spanning Clinical Pharmacology (Cluster 1), Preclinical Models (Cluster 2), and Analytical Forensics (Cluster 3), reflects the multitude of approaches used to investigate minor cannabinoids. The prominence of “cannabinoid”, “CB2”, and “nonhuman” as central terms supports the observation that cannabinoid research operates at the intersection of molecular biology and preclinical pharmacology, with increasing translation into therapeutic applications.

The temporal evolution of research themes, captured through longitudinal keyword mapping, indicates a clear progression from foundational metabolic and pharmacokinetic studies (1970–1990 s) to more recent molecular, computational, and translational approaches. The early focus on “kinetics” and “microsomes” (Grotenhermen 2003) laid the groundwork for understanding bioavailability. In the modern era (2015–2024), the emergence of terms such as “molecular docking,” “CBG,” and “CBGA” suggests a paradigm shift toward bioinformatics and systems biology. This integration of in silico modeling with wet-lab pharmacology marks a new frontier in drug discovery, allowing researchers to screen minor cannabinoids against specific targets more efficiently (Bell et al. 2024; Abbou et al. 2025).

Furthermore, the Most Cited Documents analysis confirms that the field is anchored in rigorous pharmacology rather than just recent trends. The enduring influence of Pertwee (2008) on receptor profiles and Grotenhermen (2003) on pharmacokinetics serves as the intellectual cornerstone of the discipline. These works provide the essential mechanistic definitions, agonism, antagonism, and metabolic processing that currently guide the clinical investigation of minor cannabinoids.

In summary, the evolution of the research landscape, viewed through the lens of minor cannabinoids embedded within the broader field, highlights the need to invest in translational research linking preclinical findings to human studies, expand omics-based approaches to elucidate cannabinoid mechanisms, and refine therapeutic pipelines for non-psychotropic cannabinoids.

### Study limitations

A limitation of this study is the restriction to English-language publications, which, while necessary for consistent text mining, may overlook relevant contributions from non-English publishing authors. Additionally, the analysis relies on database metadata, specifically Keywords Plus, which may lag behind the most current terminology used by authors in this rapidly evolving field. Finally, citation metrics provide a quantitative measure of scientific dissemination but do not reflect the clinical validity or methodological quality of the included studies. The findings should be interpreted as a map of research activity rather than an assessment of clinical efficacy.

## Conclusion

As the field continues to grow, it becomes increasingly important to bridge gaps in our understanding of minor cannabinoids and ensure that their full therapeutic potential is realized. By delineating the research landscape of these compounds as they are embedded within the broader cannabinoid literature, this bibliometric analysis hopes to stimulate discussion and encourage interdisciplinary collaboration among researchers working at the forefront of cannabis science.

## Supplementary Information

Supplementary Material 1

## Data Availability

No datasets were generated or analysed during the current study.

## Abbreviations

CBC: Cannabichromene
CBD: Cannabidiol
CBDA: Cannabidiolic acid
CBE: Cannabielsoin
CBF: Cannabifuran
CBFD: Cannabifuran derivative
CBG: Cannabigerol
CBGA: Cannabigerolic acid
CBLA-C5: Cannabicyclolic acid
CBL-C5: Cannabicyclol
CBLV-C3: Cannabicyclovarin
CBN: Cannabinol
CBTV: Cannabitriolvarin
CBT: Cannabitriol
CBV: Cannabinodivarin
CBVD: Cannabinodivarin derivative
CBR: Cannabirpsol
DCBF: Dehydrocannabifuran
HPLC: High-performance liquid chromatography
MS: Mass spectrometry
NMR: Nuclear magnetic resonance (spectroscopy)
PRISMA: Preferred Reporting Items for Systematic Reviews and Meta-Analyses
R: R statistical computing (software)
THC: Δ⁹-tetrahydrocannabinol
THCA: Tetrahydrocannabinolic acid
THCV: Tetrahydrocannabivarin
WOS: Web of Science
