# Supplementary material for: Mapping the research landscape of minor cannabinoids: a bibliometric analysis of research trends and hotspots
Source: J Cannabis Res. 2026 Feb 10;8:38. doi: 10.1186/s42238-026-00402-2 (PMC12990395; doi:10.1186/s42238-026-00402-2)
Supplement: Supplementary file 1 — Supplementary Material 1 [file 42238_2026_402_MOESM1_ESM.docx]

# Supplementary material

S1: The search query used for each database.

SCOPUS

TITLE-ABS-KEY ( "minor cannabinoid*" OR "rare cannabinoid*" OR "cannabigerol*" OR "CBG*" OR "cannabinol*" OR "CBN*" OR "cannabichromene*" OR "CBC*" OR "tetrahydrocannabivarin*" OR "THCV*" OR "cannabidivarin*" OR "CBDV*" OR "cannabidiolic acid*" OR "CBDA*" OR "cannabielsoin*" OR "CBE*" OR "cannabifuran*" OR "CBF*" OR "cannabicyclol*" OR "CBL*" OR "cannabitriol*" OR "CBT*" OR "cannabichromevarin*" OR "CBCV*" OR "cannabigerovarin*" OR "CBGV*" ) AND TITLE-ABS-KEY ( "cannabinoid*" OR "phytocannabinoid*" OR "endocannabinoid*" OR "cannabinoid system" OR "cannabinoid receptor*" OR "CB1" OR "CB2" )

WOS

TS=("minor cannabinoid*" OR "rare cannabinoid*" OR "cannabigerol*" OR "CBG*" OR "cannabinol*" OR "CBN*" OR "cannabichromene*" OR "CBC*" OR "tetrahydrocannabivarin*" OR "THCV*" OR "cannabidivarin*" OR "CBDV*" OR "cannabidiolic acid*" OR "CBDA*" OR "cannabielsoin*" OR "CBE*" OR "cannabifuran*" OR "CBF*" OR "cannabicyclol*" OR "CBL*" OR "cannabitriol*" OR "CBT*" OR "cannabichromevarin*" OR "CBCV*" OR "cannabigerovarin*" OR "CBGV*") AND TS=("cannabinoid*" OR "phytocannabinoid*" OR "endocannabinoid*" OR "cannabinoid system" OR "cannabinoid receptor*" OR "CB1" OR "CB2")

PUBMED

( "minor cannabinoids"[Title/Abstract] OR "cannabigerol*"[Title/Abstract] OR "CBG"[Title/Abstract] OR "CBGA"[Title/Abstract] OR "CBGV"[Title/Abstract] OR "cannabinol*"[Title/Abstract] OR "cannabinol"[MeSH Terms] OR "CBN"[Title/Abstract] OR "CBNA"[Title/Abstract] OR "cannabichromene*"[Title/Abstract] OR "CBC"[Title/Abstract] OR "CBCA"[Title/Abstract] OR "CBCV"[Title/Abstract] OR "tetrahydrocannabivarin*"[Title/Abstract] OR "THCV*"[Title/Abstract] OR "cannabidivarin*"[Title/Abstract] OR "CBDV*"[Title/Abstract] OR "cannabidiolic acid*"[Title/Abstract] OR "CBDA*"[Title/Abstract] OR "cannabielsoin*"[Title/Abstract] OR "CBE"[Title/Abstract] OR "cannabifuran*"[Title/Abstract] OR "CBF"[Title/Abstract] OR "cannabicyclol*"[Title/Abstract] OR "CBL"[Title/Abstract] OR "CBLA"[Title/Abstract] OR "cannabitriol*"[Title/Abstract] OR "CBT"[Title/Abstract] OR "cannabichromevarin*"[Title/Abstract] OR "cannabigerovarin*"[Title/Abstract] ) AND ( "cannabinoids"[MeSH Terms] OR "cannabinoid*"[Title/Abstract] OR "phytocannabinoid*"[Title/Abstract] OR "endocannabinoid*"[Title/Abstract] OR "endocannabinoids"[MeSH Terms] OR "cannabinoid system"[Title/Abstract] OR "cannabinoid receptor*"[Title/Abstract] OR "receptors, cannabinoid"[MeSH Terms] OR "CB1"[Title/Abstract] OR "CB2"[Title/Abstract] )

S2: Full list of keywords per cluster

| **Node** | **Cluster** |
| --- | --- |
| adult | 1 |
| cannabidiol | 1 |
| cannabinoid | 1 |
| cannabinoid derivative | 1 |
| cannabis | 1 |
| cb2 | 1 |
| cbc | 1 |
| cbda | 1 |
| cbdv | 1 |
| cbg | 1 |
| cbga | 1 |
| cbn | 1 |
| chemical structure | 1 |
| chemistry | 1 |
| chromatography, high pressure liquid | 1 |
| comparative study | 1 |
| controlled study | 1 |
| delta8 tetrahydrocannabinol | 1 |
| dronabinol | 1 |
| drug analysis | 1 |
| drug blood level | 1 |
| drug determination | 1 |
| drug isolation | 1 |
| drug metabolism | 1 |
| drug metabolite | 1 |
| drug screening | 1 |
| drug stability | 1 |
| female | 1 |
| gas chromatography | 1 |
| high performance liquid chromatography | 1 |
| human | 1 |
| liquid chromatography-mass spectrometry | 1 |
| male | 1 |
| mass fragmentography | 1 |
| mass spectrometry | 1 |
| metabolism | 1 |
| methodology | 1 |
| oral drug administration | 1 |
| plant extract | 1 |
| plant extracts | 1 |
| quantitative analysis | 1 |
| thc | 1 |
| thca | 1 |
| thcv | 1 |
| theoretical study | 1 |
| unclassified drug | 1 |
| animal | 2 |
| animal cell | 2 |
| animal model | 2 |
| animal tissue | 2 |
| antiinflammatory activity | 2 |
| brain | 2 |
| dose response | 2 |
| dose-response relationship, drug | 2 |
| drug effect | 2 |
| drug efficacy | 2 |
| drug mechanism | 2 |
| drug structure | 2 |
| drug synthesis | 2 |
| endocannabinoid | 2 |
| enzyme activity | 2 |
| epilepsy | 2 |
| gene expression | 2 |
| genetics | 2 |
| human cell | 2 |
| in vitro study | 2 |
| in vivo study | 2 |
| inflammation | 2 |
| mice | 2 |
| nonhuman | 2 |
| protein expression | 2 |
| rat | 2 |
| receptor, cannabinoid, cb1 | 2 |
| signal transduction | 2 |
| 11 hydroxydronabinol | 3 |
| accuracy | 3 |
| adolescent | 3 |
| blood | 3 |
| calibration | 3 |
| cannabis smoking | 3 |
| chromatography, liquid | 3 |
| extraction | 3 |
| gas chromatography-mass spectrometry | 3 |
| hair | 3 |
| limit of detection | 3 |
| limit of quantitation | 3 |
| liquid chromatography | 3 |
| marijuana smoking | 3 |
| metabolites | 3 |
| middle aged | 3 |
| procedures | 3 |
| saliva | 3 |
| sensitivity and specificity | 3 |
| solid phase extraction | 3 |
| substance abuse | 3 |
| substance abuse detection | 3 |
| tandem mass spectrometry | 3 |
| young adult | 3 |
